# Stillbirth, Neonatal, and Child Mortality in Bangladesh: Progress and Persistent Public Health Challenges

**DOI:** 10.1002/puh2.70312

**Published:** 2026-07-03

**Authors:** Md. Siddikur Rahman, Md. Abu Bokkor Shiddik

**Affiliations:** ^1^ Department of Statistics Begum Rokeya University Rangpur Bangladesh

**Keywords:** Bangladesh, child mortality, maternal and child health, Public Health Challenges, Sustainable Development Goals

## Abstract

Bangladesh made substantial gains in child survival over two decades, with the under‐five mortality rate falling from 94 to 30 per 1000 live births between 2000 and 2020. Recent data from the Bangladesh Bureau of Statistics, however, point to a troubling reversal. The infant mortality rate rose to 27 per 1000 live births in 2023, whereas neonatal mortality climbed from 16 to 20 per 1000 live births within a single year. More than 100,000 children under 5 died in 2023, with two‐thirds of deaths occurring in the first month of life. Bangladesh also records around 63,000 stillbirths annually, representing a substantial public health burden and reflecting persistent gaps in maternal and antenatal care. Contributing factors include preterm birth, limited skilled attendance at delivery, pneumonia, malnutrition, and disruptions from the COVID‐19 pandemic. Strengthening rural healthcare, expanding immunization, and improving maternal and neonatal care are essential if Bangladesh is to meet its Sustainable Development Goal (SDG) 3.2 targets by 2030.

## 1 Introduction

Bangladesh has greatly reduced child mortality rates over the last two decades. The government has drastically reduced child mortality rates through initiatives like increased immunization programs, improved prenatal care, and community‐based health projects. In 2000, the under‐5 mortality rate was 94 per 1000 births. By 2010, it had reduced to 53 per 1000 live births, and by 2020, it had fallen even lower to 30. Infant mortality decreased from 65 per 1000 in 2000 to 24 in 2020, whereas neonatal mortality improved from 41 to 16 during the same time period. These results demonstrate years of dedication to improving mother and child healthcare, backed up by strong policy and international partnerships [1, 2].

Recent statistics have raised alarm about a reversal in progress on child mortality in Bangladesh. According to the Bangladesh Bureau of Statistics (BBS), the infant mortality rate climbed to 27 per 1000 live births in 2023, reversing previous improvements. Neonatal deaths also saw an increase, rising from 16 per 1000 live births in 2022 to 20 per 1000 in 2023 [3]. This alarming trend puts Bangladesh at risk of falling short of its Sustainable Development Goal (SDG) targets for maternal and child health, specifically SDG 3.2, which aims to end preventable deaths of newborns and children under five by 2030. UNICEF reports that over 100,000 children under 5 lost their lives in Bangladesh during 2023, with more than two‐thirds of these fatalities occurring within the first month of life [4]. Compounding these losses, Bangladesh records approximately 63,000 stillbirths each year, representing a substantial public health burden and pointing to serious and unresolved gaps in maternal and antepartum care [2, 5]. These troubling developments highlight critical weaknesses within the healthcare system that require urgent attention and intervention.

### 1.1 What's Causing the Increase?

A combination of factors has contributed to this rise in child mortality. Preterm births, complications during labor, infections, and a shortage of skilled caregivers remain major challenges [6, 7]. Expanding skilled birth attendance and strengthening emergency obstetric care at district hospitals are essential responses to these gaps. Stillbirths are also a critical issue, with around 63,000 cases reported annually, highlighting persistent challenges in maternal and antenatal healthcare in Bangladesh [2, 8]. Improved antepartum surveillance, fetal monitoring, and timely referral of high‐risk pregnancies could substantially reduce preventable stillbirths [9]. Comparatively, Sri Lanka and Maldives have made remarkable progress in addressing stillbirth rates, highlighting gaps in Bangladesh's maternal healthcare system [10].

Infectious diseases, such as pneumonia, continue to pose significant threats, claiming the lives of more than 24,000 children in 2023 [11]. Alarmingly, only 60% of caregivers sought proper treatment for pneumonia, with even fewer doing so in rural areas. Scaling up childhood immunization and strengthening community‐level case management of pneumonia can meaningfully reduce these deaths. Additionally, accidental drowning is a frequently underestimated but preventable cause of death, accounting for approximately 14,000 child fatalities every year, predominantly among children aged one to four [1, 4]. Community‐based water safety programs and supervised play spaces for young children in riverine areas offer practical and proven approaches to reducing these losses.

Malnutrition is another major issue. Approximately 28% of children under five are stunted, and 9% are malnourished, making them more susceptible to sickness [12]. Community nutrition programs and micronutrient supplementation should be scaled up to address this burden. Maternal health issues, such as teenage pregnancies, anemia, and insufficient prenatal care, all raise the chance of difficulties during childbirth. Stronger adolescent reproductive health programs and increased antenatal care coverage can reduce these risks considerably. Experts believe that improved maternity and newborn care might avert up to two‐thirds of neonatal deaths.

The ongoing consequences of the COVID‐19 epidemic have worsened these issues. Routine immunizations were disrupted, and families delayed seeking medical treatment due to concerns about exposure and movement limitations. These disturbances put a strain on the healthcare system, especially in rural and marginalized communities. Urgent catch‐up immunization campaigns are needed to recover the ground lost during the pandemic. In countries like Nepal and Bhutan, improved maternal education and healthcare access have contributed to better outcomes, offering a model for Bangladesh to consider [13].

### 1.2 Regional Inequalities and Environmental Challenges

**FIGURE 1:**
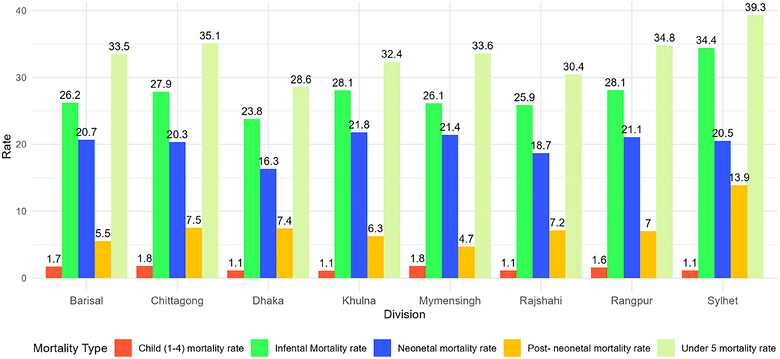
Division‐wise neonatal, postneonatal, infant, child (1–4 years), and under‐five mortality rates in Bangladesh in 2023 (per 1000 live births). *Source:* Bangladesh Bureau of Statistics (BBS), Sample Vital Statistics Report 2023 [3].

Child mortality rates vary greatly between areas in Bangladesh. Sylhet, for example, has the country's highest under‐five mortality rate, at 39.3 per 1000 live births (Figure 1) [3]. Factors such as inadequate healthcare access, poor nutrition, and regional poverty aggravate the discrepancy. Meanwhile, Dhaka has the lowest under‐five mortality rate, with an infant mortality rate of 23.8 per 1000 live births, owing to improved healthcare infrastructure and greater maternal education levels [14]. However, urban slums in Dhaka continue to pose substantial concerns due to overpopulation and poverty.

Environmental factors like flooding, contaminated water, and food insecurity also complicate the situation. Over the past 30 years, an estimated 150,000 excess infant deaths in Bangladesh have been linked to living in flood‐prone areas [15]. Climate change is predicted to exacerbate these challenges further, increasing the likelihood of waterborne diseases and food shortages that disproportionately affect children.

### 1.3 The Way Forward

Reversing the recent rise in child mortality and stillbirths will require a serious and sustained effort on multiple fronts. Strengthening healthcare infrastructure in rural and underserved areas must come first. A large share of preventable neonatal and child deaths occur in places where skilled birth attendants, functioning referral pathways, and emergency obstetric care are either absent or inadequate. Investment in district and upazila‐level health facilities, supported by community health worker programs, can substantially close this gap. Sri Lanka's experience in reducing both neonatal and stillbirth rates through community‐based midwifery and strong primary care offers an instructive model for Bangladesh to draw on [16].

Reducing the high stillbirth burden calls for dedicated antenatal surveillance. Routine antenatal visits should include fetal movement monitoring, third‐trimester ultrasound where feasible, and clear referral protocols for high‐risk pregnancies. Training community health workers to recognize warning signs of fetal compromise can allow timely intervention before loss occurs.

Immunization programs must be strengthened and extended, with priority given to communities that missed out during the COVID‐19 pandemic. Catch‐up campaigns for pneumococcal, measles, and other vaccines should be carried out using mobile units in hard‐to‐reach and flood‐prone areas.

Malnutrition requires an integrated response that goes beyond supplementation. Therapeutic feeding programs, exclusive breastfeeding promotion, and micronutrient support for pregnant and lactating women need to be delivered at scale. Tackling adolescent marriage and early pregnancy through education and enforcement of existing laws is equally important, as young maternal age is a well‐established risk factor for both stillbirth and neonatal death.

Drowning prevention deserves far more attention than it currently receives. Supervised play areas and safe spaces for young children, particularly in riverine and coastal communities, have demonstrated substantial reductions in drowning deaths in similar settings across South and Southeast Asia. Bangladesh should adopt and expand such programs without delay.

Climate resilience must be built into the health system rather than treated as an afterthought. Given that more than 150,000 excess infant deaths over the past three decades have been associated with flood exposure, health facilities need to remain functional during extreme weather events [15]. Pre‐positioned supplies, early warning systems, and community emergency preparedness training are practical starting points.

Finally, better data are needed to guide all of the above. Gaps in cause‐of‐death recording and stillbirth classification limit the government's ability to direct resources where they are most needed. Investing in civil registration systems and community‐level verbal autopsy programs will produce the evidence base required to track progress honestly toward the 2030 SDG targets.

## 2 Conclusion

In conclusion, while Bangladesh has made commendable progress in reducing child mortality over the past two decades, recent setbacks signal the need for urgent reforms. The high burden of stillbirths adds a further dimension that demands dedicated policy attention. Strengthening healthcare systems, addressing disparities in access to care, and prioritizing maternal and neonatal health are essential to reversing these trends. By implementing targeted reforms and learning from the successes of other South Asian nations, Bangladesh can work toward meeting its goals and securing a healthier future for its children.

## Author Contributions

**Md. Siddikur Rahman**: conceptualization, literature review, data analysis, and writing the main manuscript. **Md. Abu Bokkor Shiddik**: literature review and data analysis.

## Funding

The authors have nothing to report.

## Ethics Statement

The authors have nothing to report.

## Consent

The authors have nothing to report.

## Conflicts of Interest

The authors declare no conflicts of interest.

## Data Availability

The datasets used and/or analyzed during the current study are available from the corresponding author on reasonable request.
